# Reversibly switching water droplets wettability on hierarchical structured Cu_2_S mesh for efficient oil/water separation

**DOI:** 10.1038/s41598-019-48952-1

**Published:** 2019-08-28

**Authors:** Shanya Xu, Rui Sheng, Yali Cao, Junfeng Yan

**Affiliations:** Institute of Applied Chemistry, Xinjiang Key Laboratory of Energy Materials Chemistry, Ministry of Education, Xinjiang University, Urumqi, 830046 China

**Keywords:** Surface assembly, Nanowires

## Abstract

Surfaces with reversible wettability have broad applications but remain challenging since the switching process is usually energy intensive and complex. In this paper, a pyramid shaped Cu_2_S film with hierarchical micro/nanostructures is formed on a commercial copper mesh. This film is formed by a spontaneous redox sulfuration reaction and results in a roughened surface, which enables reversible wetting transition between superhydrophilicity to superhydrophobicity. This switching occurs by simple processes such as alternately storing in air or using an ethanol solution treatment and yields cyclic wettability switching for many cycles. This convenient wetting transition behavior, as well as strong stability and efficient oil/water separation with efficiency exceeding 98%, renders it as a potentially useful mesh material for switchable surfaces.

## Introduction

In recent years, the increase in oil leaks and spills requires industrial technology to efficiently separate oil/water immiscible mixtures in mild conditions^1–13^. Among different separation technologies that are applicable to industrial processes as well as our daily life^14,15^, membrane-shaped materials, such as mesh^16–22^, fabric^23–26^, and membrane materials^27–29^, have been gained widespread interest due to higher separation efficiency and probable use in large scale manufacturing^30–34^. Since surface wettability of these textured membranes can dramatically affect water or oil droplet adhesion between substrates, and hence affect the oil/water separation process^35–39^, designing and preparing wettability switchable membranes would be a convincing way to enable the separation process on demand^40–45^.

Surface morphology, or roughness, along with the chemical composition of a material combine to determine the degree of wettability of a material. A material that is naturally hydrophilic or hydrophobic can be taken to a degree of superhydrophilicity or superhydrophobicity through hierarchial morphology or a micro-structured topography. Taking inspiration from nature, materials can have tailored wettability by patterning chemical composition or surface structure. A series of superwetted surfaces can be designed and created for use in applications such as patterned wetting, anti-fouling, self-cleaning, petroleum industry for oil/water separation, *et al*.^46–48^.

The hierarchical micro/nanoscale structures and chemical composition on surfaces is responsible for the superwetting behaviors^49–57^, which enables the oil/water separation process to be efficient and stable. For example, the Feng group fabricated a hierarchical polytetrafluoroethylene coated mesh to allow oil to permeate the film while water was blocked due to the superhydrophobicity and superoleophilic properties^58^. From this idea, different chemical substances can be coated on a roughened surface, or mesh, creating tunable superhydrophobic or superoleophilic properties that can be controlled via morphology engineering. The chemical composition or the components of solid surfaces is all-important in controlling the intermolecular forces between a solid and a liquid, which can be triggered by a series external stimuli such as light, temperature, solvents, pH, electrical potential, and ions or molecules^41,42,59–62^. Using these external stimuli to switch wettability is reversible over many cycles and therefore this is a robust method for realizing wettability changes on a surface^63–68^. For example, Guo *et al*. assembled functional organic molecules on copper mesh that was superhydrophobic in acidic solutions due to its pH-responsive property, but can be converted to be superhydrophilic as well as superoleophobic in submerged alkaline solutions just by tailoring the pH. The protonation and deprotonation processes of functional groups allow access to separate oil/water bidirectionally^69^.

**Figure 1:**
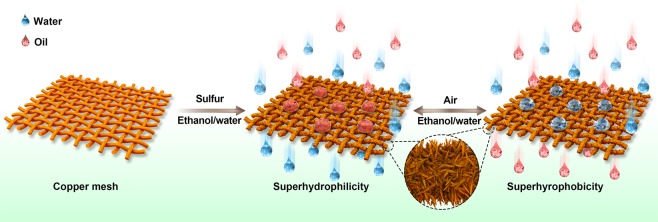
Schematic illustration of fabrication process of Cu_2_S-copper mesh materials and the usage for controlled separation of different oil/water mixtures.

In recent years, besides surface coating of active materials and chemically bonded molecules on mesh^70,71^, *in-situ* grown micro-nanostructured porous materials have received tremendous amounts of attention due to superior mechanical performance and better flexibility^72–75^. Copper mesh, as a typical engineering material, has micro/nano-structures that can be fabricated on the surface to enable special wettability. However, matchstick-like nanostructured Cu_2_S copper mesh can turn from superhydrophilic to superhydrophobic after two weeks of storage in air without any modification^76^. The general strategy is to create diverse micro/nanostructures on a substrate and then engineer the surface chemical composition regulation to dynamically build smart surfaces with controlled wettability and hence control the separation of oil/water mixtures^74^. In addition, after the oil/water separation is complete, the wettability of the fabricated mesh can be transitioned back using UV light or temperature. In order to simplify the fabrication process and wetting regulation method conveniently, as well as promote reliable stability, we propose a sulfuration approach to fabricate hierarchically pyramid-shaped micro/nanostructured Cu_2_S layers on copper mesh, as shown in Fig. 1, on which the oil/water separation process is triggered via external stimuli such as air or ethanol solution. Obviously, the commercially available copper mesh possessed smooth surfaces but can become roughened with pyramid-like micro-structures after sulfuration. When the mesh exhibits superhydrophilicity, water droplets can wet the surface and enable water to continuously permeate the structure, while blocking light oils. However, after storing in air for 2 days, the mesh can completely flip to become superhyrophobic hence allowing heavy oils to pass through the films while repelling water. This transition can be reversibly switched many times to achieve on-demand separation of oil/water mixtures with good yield through immersion in ethanol for four hours and leaving it in air.

## Results

### Morphology of Cu_2_S films

**Figure 2:**
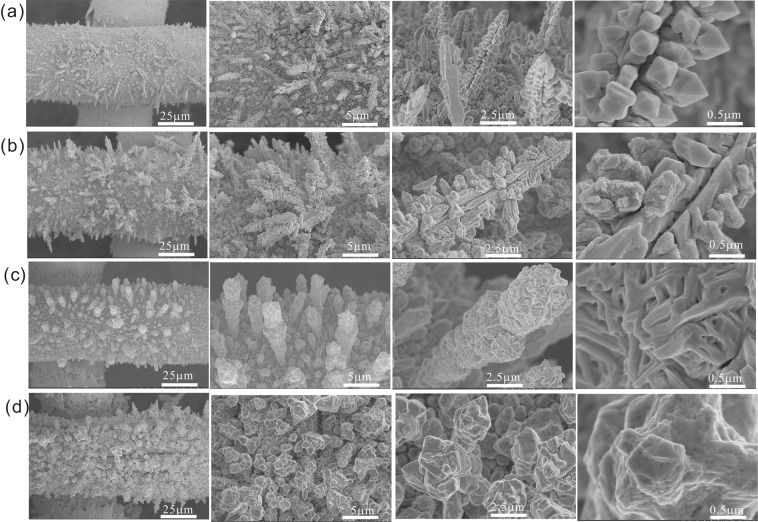
SEM images of Cu_2_S-copper mesh at different sulfuration durations: (**a**) 30 min, (**b**) 60 min, (**c**) 90 min, and (**d**) 120 min. Scale bars in (**a**–**d**) are 25 μm, 5 μm, 2.5 μm, 0.5 μm, respectively.

The cleaned copper mesh was immersed into a sulfur solution prepared with a mixture of 2:1(volume) ethanol and water, where vertically aligned micro/nanoscale hierarchical structures with a pyramid shape grew on copper mesh after the reaction proceeded for about 30 minutes at 65 °C. These structures had an average diameter and length of 200–400 nm and about 7 μm respectively, as depicted in the scanning electron microscopy (SEM) images in Fig. 2(a). The purchased purple copper mesh features smooth surfaces with no obvious micro/nanometer-scale structures, but can become dark grey with considerable roughness after full and even sulfuration (see Fig. S1(b,c)). In contrast, the high-resolution SEM image revealed that larger clustered pyramid micro-structures grew on the mesh surface upward and horizontally when the sulfuration time was increased to 60 min (Fig. 2(b)). The aligned micro/nanoscale structures gradually disappeared when prolonged etching times were used for 90 min and 120 min. The longer times caused the vertically aligned pillar-arrayed architectures to possesses considerable roughness at the micrometer scale that formed gradually as shown in Fig. 2(c,d). The vertically oriented Cu_2_S micropillars that grew on the copper mesh were confirmed by XRD measurement (Fig. S2(a)), the characteristic peaks at 2θ equal to 27.5, 31.9 and 45.8 are strongly associated with the (111), (200), and (220) planes of Cu_2_S with a cube phase (JCPDS No. 65–2980). In addition, the microstructure of Cu_2_S materials as well as the local atomic arrangements can be observed through Raman spectroscopy. From the Raman spectrum the characteristic peak of Cu_2_S can be observed at 469 cm^−1^ (Fig. S2(b)). Energy Dispersive Spectroscopy (EDS) shows that the mass ratio of Cu:S is nearly 2:1, which further proves the successful Cu_2_S formation on the copper mesh, Figure S2(c).

### Wettability behavior of Cu_2_S

**Figure 3:**
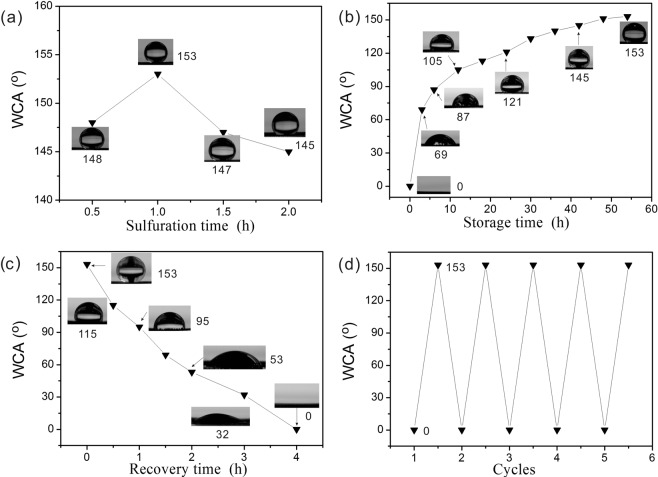
(**a**) WCA of the Cu_2_S copper mesh with different sulfuration times after storage in air for 2 days. The reversible wetting transition of Cu_2_S copper mesh with 60 min sulfuration: (**b**) Variation of WCA of initially prepared samples when stored in air for different times; (**c**) Variation of WCA for superhydrophobic Cu_2_S copper meshes when immersed in ethanol solution for different times; (**d**) Reversible wettability change of the Cu_2_S mesh between superhydrophobicity and superhydrophilicity.

The wettability of Cu_2_S-copper mesh generally depends on the surface morphology directed by roughness and its chemical composition. The commercially available original copper mesh has a water contact angle (WCA) of 102° for Wendell state, and the underwater/oil contact angle (OCA) was 138° (Fig. S1(a)). The initially prepared fresh Cu_2_S-copper mesh (prepared with 60 min sulfuration time) maintains superhydrophilic properties where the water droplet can fully wet the surfaces, giving the increased WCA of 69° after exposure to air for 3 hours. Prolonged storage time of 6 hours demonstrated that the surface nearly turned hydrophobic as demonstrated with the WCA of 87°, with longer storage of over two days enabling the full transition to superhydrophobicity and a WCA of 153° (Fig. 3b). Figure 3a shows that all pyramid-like Cu_2_S samples possess hydrophobicity after sulfuration for different times simultaneously. The WCA recorded in these measurements demonstrated that the surface with 60 min of sulfuration has a higher degree of hydrophobicity with a higher contact angle of 153°, which can be ascribed to the greater degree of hierarchical micro/nanoscaled structures and this sample was selected for all tests. Interestingly, a water droplet WCA of 115° appeared on the mesh surfaces after 30 min, after they were immersed in water ethanol solution for approximately 4 hours and drying in air. The mesh can switch to hydrophilic after further storing in air for 3 hours, and gradually switch to superhydrophilicity after 4 hours as displayed in Fig. 3c, which suggests a convenient and simple method to switch water droplet wettability with good controllability. Significantly, this wettability transition can be reversibly switched for many times as shown in Fig. 3d, the superhydrophilic mesh can spontaneously turn to superhydrophobic after storing in air for two days, and can turn back after ethanol solution treatment for 4 hours, allowing continuous modulation of superwettability via a simple way. This unique reversible wettability transition is useful for a variety of oil/water separations on demand. Subsequently, in order to test the corrosion resistance of the Cu_2_S membrane in an aqueous environment, a series of experiments was performed where the freshly prepared Cu_2_S mesh was immersed in 1 M HCl, 1 M NaOH, and 1 M NaCl aqueous solutions for 24 hours, respectively. Oil contact angle (OCA) tests show that underwater superoleophobic angles of all samples were 145–155°, which completely indicate the Cu_2_S membrane could withstand several aqueous chemicals (Fig. S3).

**Figure 4:**
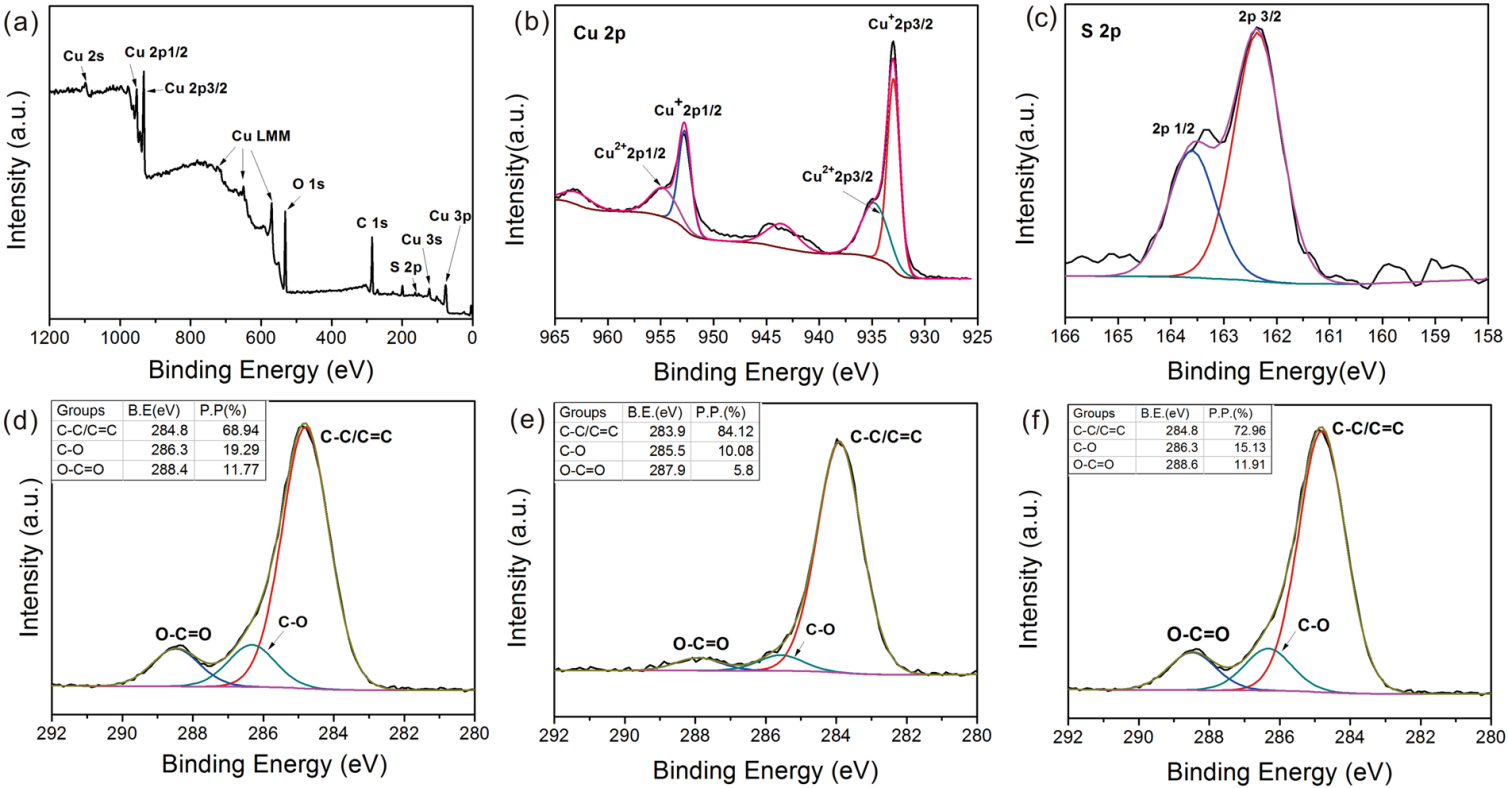
XPS spectra of Cu_2_S-mesh (**a**), core level Cu 2p (**b**), core level S 2p (**c**). C 1 s core level XPS spectrum of the freshly prepared superhydrophilic Cu_2_S film (**d**), superhydrophobic Cu_2_S film: stored in air for two days (**e**), superhydrophilic Cu_2_S film: soaking in ethanol solution for 4 hours (**f**).

The variation in chemical composition with time for the Cu_2_S-mesh from 60 min sulfuration can be analyzed and identified by X-ray photoelectron spectroscopy (XPS). The survey scan spectrum shows that Cu, O, S, and C were present (Fig. 4a). The Cu 2p_3/2_ and Cu 2p_1/2_ peaks are located at binding energies of 932.5 and 952.8 eV can be assigned to the Cu^+^ species, and the existence of Cu^2+^ species can be confirmed from the Cu 2p_3/2_ and Cu 2p_1/2_ doublet peaks at 934.8 and 954.9 eV. Satellite peaks at binding energies of 943.9 and 963.4 eV prove the presence of Cu^2+^ (Fig. 2c). The S 2p can also be divided into two peaks for 2p_3/2_ and 2p_3/1_ at the binding energies of 162.3 and 163.5 eV (Fig. 2c). High resolution XPS of C 1 s can provide the proportion of hydrophobic and hydrophilic groups on Cu_2_S mesh that most likely originate from the presence of ethanol, which can dictate the surface composition variation as well as the mechanism of wettability transition. C 1 s XPS spectrum can fully characterize the variation of the surface chemical composition during the wettability switch. Three C 1 s peaks, with binding energies of 284.8, 286.3, and 288.6 eV, correspond to C−C/C=C, C−O, and O−C=O, respectively. Meanwhile, there are possibly some hydrophobic hydrocarbons generated during the reaction process due to the presence of C−C/C=C that induce the wettability transition^76,77^. The proportion of the hydrophobic hydrocarbons was 68.94% for initial Cu_2_S-mesh, the proportion of 19.29% and 11.77% corresponding to hydrophilic groups of C−O and O−C=O, respectively (Fig. 4d). For comparison, the proportion of C−C/C=C for stored Cu_2_S-mesh after two days was 84.12%, and the proportion of C−O and O−C=O was 10.08% and 5.8%, respectively, which can possibly explain the hydrophobic properties (Fig. 4e). Furthermore, ethanol triggered reversibly switching the mesh surface to be superhydrophilic and can also be proven by XPS. The proportion of the hydrophobic hydrocarbons was decreased to 72.96%, but the proportion of hydrophilic groups C−O and O−C=O was 15.13% and 11.91%, respectively (Fig. 4f).

**Figure 5:**
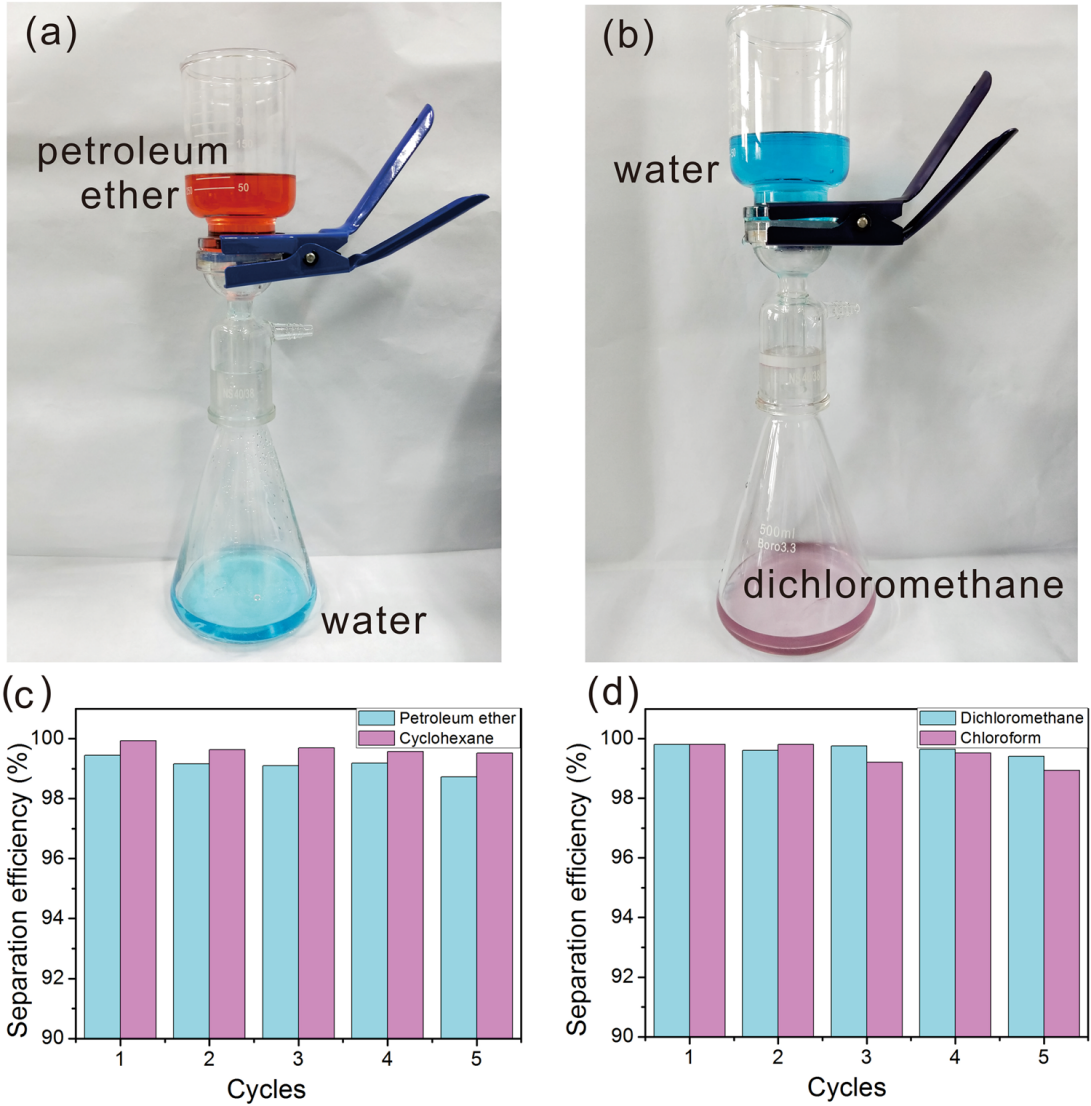
Digital photo of experimental setups of (**a**) Light oil/water separation; (**b**) heavy oil/separation. Diagram of separation efficiency and cycle number of superhydrophilic copper mesh (**c**); superhydrophobic copper mesh (**d**).

This switchable Cu_2_S copper mesh can be used for oil/water separation on-demand, and Fig. 5(a) shows digital photos of the separation process of 1:1 petroleum ether/water mixtures and 1:1 cyclohexane/water mixtures using freshly prepared Cu_2_S copper mesh. The permeation of petroleum ether could be blocked on the water-prewetted Cu_2_S mesh while water permeated through it quickly and was collected in a conical flask. The superhydrophilic Cu_2_S mesh with micro/nanoscale structures could adsorb water molecules in solution to generate superoleophobic surfaces under water while in contact with oils. In addition, superhydrophobic Cu_2_S mesh can be used to achieve heavy oil/water separation. In this case, dichloromethane (or chloroform) represented the heavy oil and it can fill into the gap of the pyramid-shaped Cu_2_S mesh, then those pockets of oil, rather than air, can repel water droplets so oil can pass through the mesh favorably (Fig. 5(b)). The ultra-low adhesion of water droplets on superhydrophobic mesh was proven by a touching-detaching experiment, whereas the structural surfaces can repel water droplets (Fig. S4). Figure 5(c,d) show the separation efficiency of the various oils with different densities, which is maintained at 98% after 5 cycles, indicating efficient separation with good stability. The formula for the separation efficiency is shown in the equation below:

$$Es=\frac{{M}_{filtrate}}{{M}_{mixture}}\times 100 \% $$

M_filtrate_ are the mass of the collected oil filtrate or water filtrate,respectively; M_mixture_ are the mass of oil or water in initial mixture solution, respectively.

## Conclusion

In summary, a Cu_2_S film with pyramid-like micro/nanoscale structures was formed on a copper mesh through a redox sulfuration synthesis. The initially prepared Cu_2_S copper mesh showed stable superhydrophilicity, with a switch to superhydrophobicity when exposed in air for two days. This reversible superwetting transition between superhydrophilicity and superhydrophobicity was simply regulated upon air or ethanol solution treatment and can be repeatedly switched for selective oil/water separation for many cycles. This hierarchical superwettable Cu_2_S film has excellent resistance to acidic or alkaline solutions, a tunable surface wetting transition and strong stability, making it ideally suited for on-demand oil/water separation by to oil density.

## Methods

### Material

Ethanol (99.7%), cyclohexane (99.5%), dichloromethane (99.5%), chloroform (99.5%), and petroleum ether (98.0%) were purchased from Tianjing Guangfu Technology Development Co. Ltd. and were used as received. Copper mesh (300 mesh size) was obtained from a local market. Sublimed sulfur (99.5%) was purchased from Tianjin Kemeng Chemical Industry Trade Co. Ltd. Methylene Blue (98.0%) and Sudan III (99%) were purchased from Shanghai Titan scientific Co. Ltd. Sodium chloride (99.5%), hydrochloric acid (37%) and sodium hydroxide (99.6%) were purchased from Tianjin Zhiyuan Chemical Reagent Co. Ltd.

### Fabrication of Cu_2_S Film on Copper Mesh

Copper mesh was cut into 5 cm ×5 cm pieces and were sequentially washed during sonication with acetone, ethanol and deionized water for 5 min. Then, 0.012 g of sulfur powder was dissolved in 150 mL ethanol/water solution (ethanol: water = 2:1), and the copper mesh was immersed in the prepared sulfur solution at 65 °C for different durations, the resulting mesh was rinsed with deionized water and dried in air. Copper undergoes oxidation reaction to form Cu^+^, and sulfur undergoes reduction reaction to form S^−2^. Low sulfur metal sulfide is formed on the surface of the copper mesh due to weak oxidizing of sulfur. The reaction process is shown: 2Cu + S→Cu_2_S.

### Switching process of Cu_2_S film

The as-prepared Cu_2_S copper mesh possesses good underwater superoleophobic property (157°), so light oil/water mixture can be efficiently separated. After storage in air for two days, superhydrophobic Cu_2_S copper mesh can separate heavy oil/water. After immersing the superhydrophobic membrane in ethanol solution (ethanol: water = 2:1) for 4 hours, the superhydrophobic films become superhydrophilic. The freshly prepared Cu_2_S mesh with superhydrophilicity was soaking in 1 M HCl, 1 M NaOH and 1 M NaCl solution for 24 hours to measure the stability.

### Characterization

The surface morphology of the sample was observed by Scanning electron microscopy (SEM, S-4800, HITACHI, Japan). The crystalline state and composition of materials were determined by X-ray diffractometry (Bruker, D8 advance diffractometer) and Raman spectroscopy (Raman, SENT-ERRA, Bruker, Germany). Surface elements were analyzed by X-ray photoelectron spectroscopy (Thermo Fisher Scientific Escalab 250 Xi system operated at 15 kW with a monochromatic Al Kα source.) The contact angle of these samples was measured using a contact angle measurement system (JJ-2000B2) by dropping a small volume of liquid (~6 μL) from a 1-mL micrometer syringe onto the target surface. At least three measurements were performed on each sample. The typical error in measurements was ±2°.

### Oil-water separation

Oil/water separation was carried out with a designed set-up in Fig. 5, the oil/water mixture (1:1 volume) was flowing past the Cu_2_S copper mesh due to gravity and the separated liquid was eventually collected in a conical flask. Water and oil (including cyclohexane, petroleum ether, dichloromethane, and chloroform) were colored with methylene blue and Sudan III, respectively. The separation time for 200 mL mixture solution was about 30 seconds.

## Supplementary information

Supporting Information
