# Supplementary material for: Reversibly switching water droplets wettability on hierarchical structured Cu2S mesh for efficient oil/water separation
Source: Sci Rep. 2019 Aug 28;9:12486. doi: 10.1038/s41598-019-48952-1 (PMC6713748; doi:10.1038/s41598-019-48952-1)
Supplement: Supplementary file 1 — Supporting Information [file 41598_2019_48952_MOESM1_ESM.docx]

**Supporting Information**

**Reversibly switching water droplets wettability on hierarchical structured Cu_2_S mesh for efficient oil/water separation**

Shanya Xu, Rui Sheng, Yali Cao, Junfeng Yan*

Institute of Applied Chemistry, Xinjiang University, Urumqi 830046, Xinjiang

Key Laboratory of Energy Materials Chemistry, Ministry of Education, Institute of Applied Chemistry, Xinjiang University, Urumqi 830046, Xinjiang


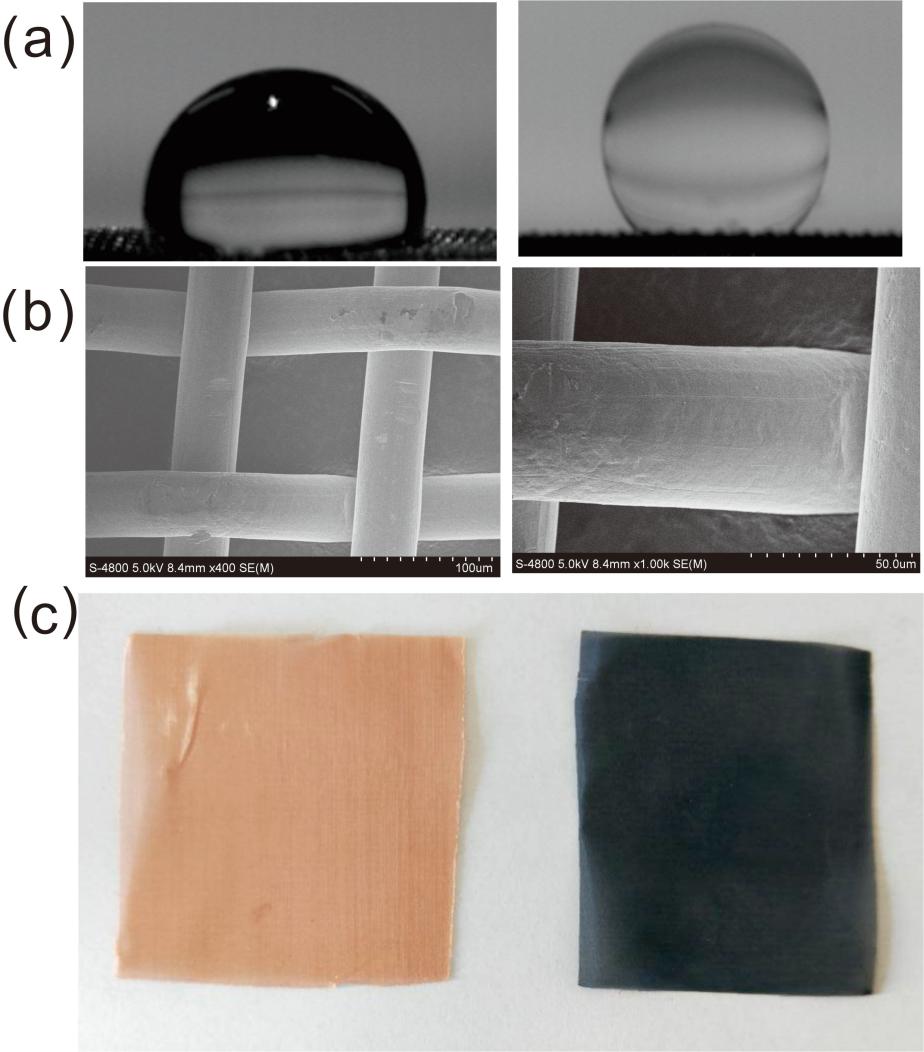


**Figure S1.** (a) The WCA of the original copper mesh, underwater oleophobic angle of the original copper mesh, (b) SEM images of the original copper mesh (25 μm), SEM images of the original copper mesh (50 μm) (c) photo of original copper mesh and after sulfuration.


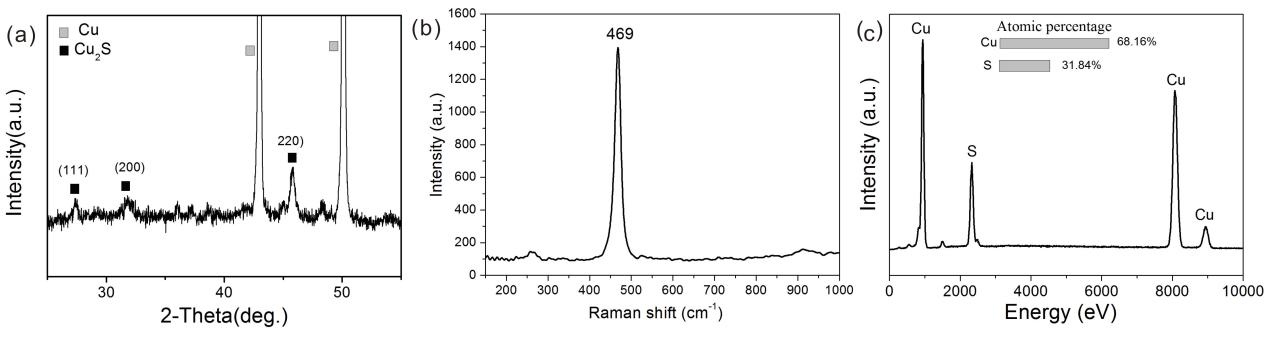


**Figure S2.** (a) XRD patterns of Cu_2_S mesh; (b) Raman spectra of the Cu_2_S mesh; (c) EDS spectra of Cu_2_S mesh.

**
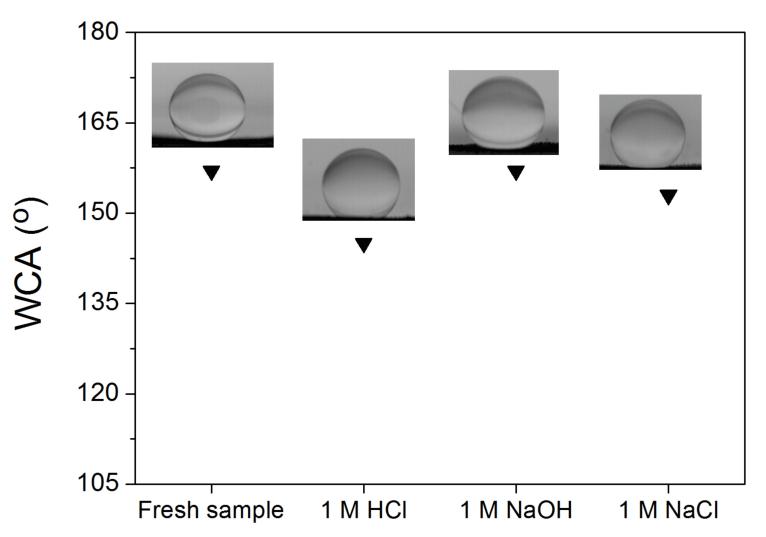
**

**Figure S3.** Underwater OCA of freshly prepared Cu_2_S mesh with superhydrophilicity after soaking for 24 h in 1 M HCl, 1 M NaOH, and 1 M NaCl solution, and the corresponding SEM images.

Freshly prepared Cu_2_S filter soaked in a corrosive medium of 1 M HCl, 1 M KOH, 1 M NaCl for 24 hours, respectively. Oil contact angle (Figure S1) in water were measured to evaluate the wetting properties of the filter. The morphologies and chemical composition of the copper mesh were analyzed using scanning electron microscope (SEM) (Figure S2). EDS spectra show the atomic percentage for the mesh surface. Copper and oxygen ratio close to 2:1, explain that the material generated on the surface of the copper mesh is Cu_2_S.


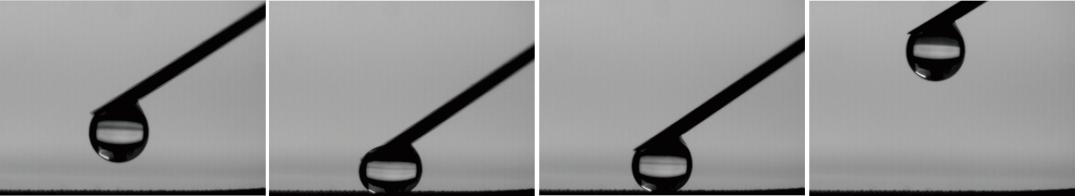


**Figure S4.** An water droplet as a probe to evaluate the adhesion of Cu_2_S.
